# Late diagnosis of medial condyle fracture of the humerus with rotational displacement in a child

**DOI:** 10.1007/s10195-011-0155-x

**Published:** 2011-08-31

**Authors:** Kwang Soon Song, Kirti Ramnani, Chul Hyun Cho, Eun Seok Son

**Affiliations:** Department of Orthopedic Surgery, School of Medicine, Keimyung University, 194 Dongsan-dong, Joong-gu, Daegu 700-712 Korea

**Keywords:** Late diagnosis, Open reduction, Medial condyle, Humerus, Child

## Abstract

For displaced medial condyle fractures in children, open reduction with internal fixation seems to be most popular treatment method. The major complication of this method is failure to make the proper early diagnosis. Corrective supracondylar humeral osteotomy has been preferred to open reduction and internal fixation for managing malunited fragments. We report a case of a child with nonunion of the medial condyle of the humerus who was subsequently treated successfully with open reduction and internal fixation.

## Introduction

Fracture of the medial condyle of the humerus in a child is rare. Kilfoyle classified these fractures into three types according to the degree of displacement. He achieved favorable results with open reduction and internal fixation even in complete and displaced fractures (type 3) by diagnosing them early [1]. Many authors have reported uniformly poor outcome for these fractures when diagnosed late and treated with open reduction and internal fixation [2–5]. We report the case of a child with nonunion of the medial condyle of the humerus with restricted and painful elbow range of motion (ROM) and cubitus varus deformity who was treated successfully with open reduction and internal fixation.

## Case report

**Fig. 1:**
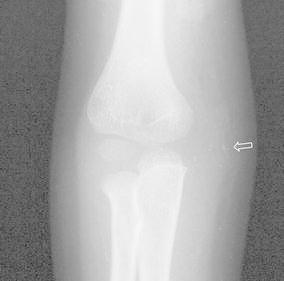
Anteroposterior radiograph of right elbow obtained on the day of injury demonstrates no fracture line; however, there are tiny radiodense shadows (*arrow*) quite distant from the bony trochlea

A 4-year 7-month-old boy fell from a height of approximately 1 m, injuring his left elbow. The physician who initially examined the child gave a provisional diagnosis of elbow sprain, having found no fracture line on anteroposterior (Fig. 1) and lateral radiographs. The elbow was immobilized in 90° flexion and neutral position for 2 weeks with long-arm splint, and then active elbow motion was encouraged. Despite physiotherapy, ROM did not improve and partial stiffness persisted. Progression of cubitus varus deformity was noted, and the boy reported constant pain during elbow motion.

**Fig. 2:**
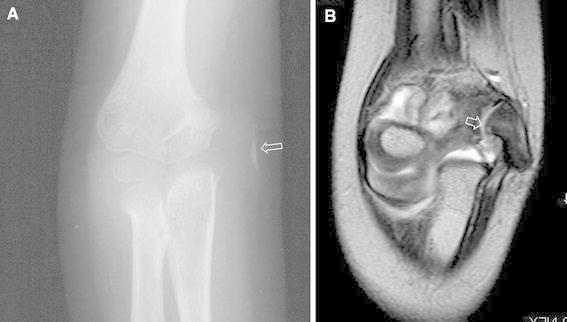
At 4 months after the initial injury, an anteroposterior radiograph showed a very thin sliver of ossification, slight narrowing of the humeroulnar joint, and mild varus alignment of the humeroulnar angle. **a** T2 weighted spin-echo magnetic resonance imaging (repetition time/echo time 4,000 ms/96 ms) showed complete rotation of the cartilaginous fragment (*arrow*) with trochlear defect (**b**)

**Fig. 3:**
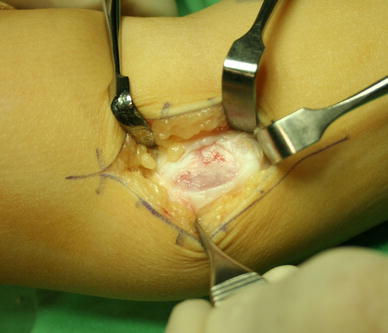
Intraoperative image shows the intramedullary cancellous surface facing outward just under the subcutaneous tissue due to complete rotation of a fragment of the cartilaginous trochlea

**Fig. 4:**
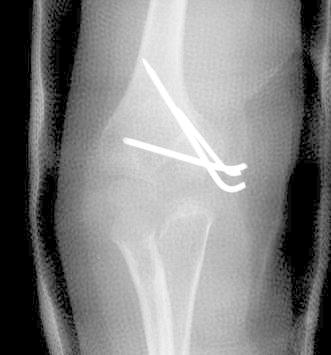
Postoperative anteroposterior radiograph. The cartilaginous trochlea was fixed with three smooth Kirschner wires. A long-arm cast was applied with the elbow fixed at 90° and the forearm in neutral position, and then was removed after 6 weeks

The patient was referred to our hospital 4 months after the initial injury. Clinical examination showed a cubitus varus deformity with decreased Baumann angle (3°) and restricted ROM: extension lag 45°; flexion 110°; arc of motion 65°. There were neither neurologic nor circulatory deficits. An anteroposterior radiograph showed a very thin sliver of ossification quite distant from the bony trochlea, slight narrowing of the humeroulnar joint, and Baumann angle 5° (Fig. 2a). Magnetic resonance imaging showed complete rotation of the cartilaginous fragment, with a trochlear defect (Fig. 2b). We decided to reduce the fracture using the open method after obtaining consent from the boy’s parents. An incision was made directly over the displaced sliver fragment. Intraoperatively, we found the intramedullary cancellous fracture surface facing laterally outward just under the subcutaneous fatty tissue (Fig. 3). The ulnar nerve was identified and protected with an elastic rubber drain. The proximal fragment was freshened with thorough curettage of the defect where the displaced distal fragment was located previously and had become filled with fibrotic tissue. We identified the medial margin of the articular cartilage and medullary space of the host bone, and reduction of the rotated fragment was accomplished without takedown of the attached flexor muscles. However, the fracture could not be reduced anatomically because the fracture surface and shape had already changed due to time elapsed since injury. We reduced the fragment in the most plausible position primarily with approximation of articular margin, fixed it with three smooth Kirschner (K) wires (Fig. 4), then repaired the soft tissue with absorbable synthetic suture material. A long-arm cast was applied with the elbow at 90° and the forearm in neutral position for 6 weeks. We removed the K wires 6 weeks after surgery under local anesthesia.

**Fig. 5:**
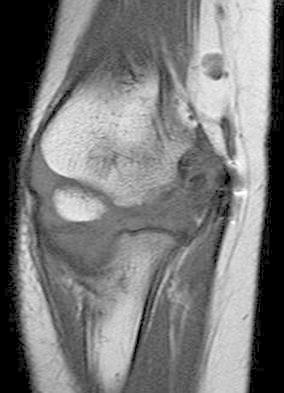
Six months after surgery, a T1-weighted spin-echo magnetic resonance imaging (repetition time/echo time 820 ms/96 ms) showed a well-positioned and united cartilaginous trochlea

Immediately after surgery, ulnar nerve palsy was noted, which recovered completely by 8 weeks after surgery with simple observation. At the 6-month follow-up, magnetic resonance imaging (MRI) showed that the cartilaginous trochlea was united and well positioned (Fig. 5).

**Fig. 6:**
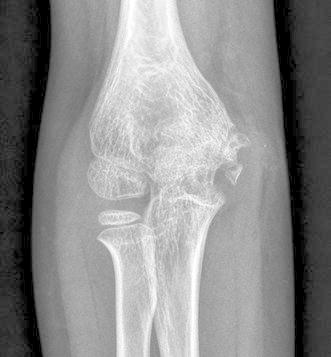
Follow-up at 3 years 2 months: anteroposterior radiograph shows an irregular, speculated bony trochlea and a normal Baumann angle (15°)

At 3 years 2 months follow-up, an anteroposterior radiograph showed an irregular, spiculated, bony trochlea and a normal Baumann angle (15°) (Fig. 6). Examination showed an improved ROM, with an extension lag of 10° and flexion to 140°; arc of motion was 130°. We obtained permission from the patient’s parents and approval from our institutional review board (Log No. 10–89).

## Discussion

Fracture of the medial condyle of the humerus is a rare injury in children, comprising 1–2% of all pediatric elbow fractures [2, 5, 6, 8]. Diagnosing such fractures radiologically can be difficult in younger children because the fracture is primarily through the cartilage, and the trochlea is not completely ossified until around the age of 9 years [2, 6, 7]. Pain, swelling, ecchymosis over the medial elbow, and radiographic findings of “a few radiodensities” far medial to the elbow are important diagnostic clues to this fracture. If this constellation of signs and symptoms is seen, other diagnostic modalities, such as MRI, ultrasonograph, arthrogram, and computed tomography are indicated. Several researchers have reported a high risk of avascular necrosis in surgical treatment of nonunion medial condylar fractures because takedown of the forearm flexor is required to expose the fracture site [1–3]. Better functional results are achieved by accepting a nonunion than by the acquired avascular necrosis [2]. Corrective supracondylar osteotomy has been considered a preferred alternative to open reduction and internal fixation of the nonunited fragment to correct the deformity and improve elbow ROM in patients in whom the fracture is discovered late [8]. We had favorable results with open reduction and internal fixation, with no serious complications. Although it is difficult to draw conclusions from one case only, we believe that leaving the flexor muscle attached to the fracture fragment is important to prevent trochlea avascular necrosis. As medial condyle fractures are intra-articular Salter Harris type IV injuries, early diagnosis is extremely important to obtain good result without serious complications [2, 7, 9]. Therefore, if there is clinical suspicion of medial condyle fracture, the patient must be further evaluated for early fracture detection.
